# The Spinning Manny Indexed Overlay System for VMAT total body irradiation

**DOI:** 10.1002/acm2.70350

**Published:** 2026-01-15

**Authors:** Lawrie Skinner, Eric Simiele, Zi Yang, Caressa Hui, Ignacio Romero, Michael Binkley, Richard Hoppe, Susan M. Hiniker, Nataliya Kovalchuk

**Affiliations:** ^1^ Department of Radiation Oncology Stanford University Stanford California USA; ^2^ Department of Radiation Oncology The University of Alabama at Birmingham Birmingham Alabama USA; ^3^ Department of Radiation Oncology University of California, Irvine Irvine California USA

**Keywords:** IMRT TBI, rotational platform, Spinning Manny, VMAT TBI

## Abstract

**Purpose:**

While modern intensity‐modulated techniques for total body irradiation (TBI) offer superior organ sparing and improved dose coverage compared to conventional TBI, their complexity has limited widespread adoption. Specifically, when treating individuals with Volumetric Modulated Arc Therapy (VMAT‐TBI), a transition between the head‐first‐supine (HFS) and feet‐first‐supine (FFS) orientations is typically required. This study introduces and evaluates the Spinning Manny Indexed Overlay System, an in‐house developed rotational platform designed to streamline VMAT‐TBI treatments.

**Methods:**

The Spinning Manny platform is a carbon fiber overlay that indexes securely to the treatment table, allowing for accurate and reproducible patient rotation. Weight‐bearing assessments, isocenter reproducibility evaluation, and dosimetric characterization through CT imaging and attenuation measurements were made. End‐to‐end testing was performed using anthropomorphic and solid water phantoms, with dosimetric verification using film and thermoluminescent dosimeters (TLDs). The system was clinically implemented for VMAT‐TBI at Stanford University. Workflow efficiency and dose delivery accuracy were assessed through in vivo dosimetry and treatment plan comparisons.

**Results:**

Commissioning tests confirmed the mechanical stability and dosimetric integrity of the Spinning Manny platform. Weight‐bearing tests demonstrated support up to 159 kg, and isocenter reproducibility after rotation was within 1 mm. Attenuation at 6 MV was measured to be 4.3% and 1.3% at beam angles 30° and 90°, respectively, relative to the couch surface. For 10 MV, the attenuation was 3.4% and 1.0% at the same angles. Gamma passing rates in the end‐to‐end tests were 96.9% (3%/2 mm) for the lung regions and 92.6% (5%/3 mm) for the VMAT/AP/PA matchline. Clinical implementation across 136 patients (age range: 1–64 years old, height range: 83.6–197.3 cm) demonstrated efficient workflow integration, with 83% requiring HFS‐to‐FFS transitions. In vivo dosimetry confirmed accurate dose delivery at the matchline (96.1% ± 5.5% relative to prescription dose).

**Conclusions:**

The Spinning Manny Indexed Overlay System effectively addresses a key logistical barrier in VMAT‐TBI by enabling accurate and reproducible HFS‐to‐FFS transitions. Its mechanical stability, low attenuation, and high isocenter reproducibility support its clinical reliability, with successful implementation across patients of varying sizes and ages. By sharing this design, we aim to support the expansion and access of VMAT‐TBI worldwide.

## 1 INTRODUCTION

Total body irradiation (TBI) has been an important component of myeloablative and nonmyeloablative conditioning regimens for allogeneic hematopoietic stem cell transplantation (HSCT) for decades. Historically, TBI has been delivered with a two‐dimensional (2D) technique at extended distances with patients in a standing, seated, or lying position. Technological advancements in planning and delivery have prompted a transition to modern intensity‐modulated techniques such as Volumetric Modulated Arc Therapy (VMAT‐TBI) and helical TomoTherapy (Tomo‐TBI).^1^ These techniques provide superior sparing of organs at risk while improving dose coverage to the target^2^ which results in reduction of radiotherapy‐related toxicities without compromising the effectiveness of TBI.^3^ Despite the benefits of VMAT‐TBI, the increased complexity in patient setup and treatment planning has limited its adoption, with only a few institutions currently utilizing this technique.^4^ Although some of the existing literature has been focused on pediatric patient populations, complexity in delivering VMAT‐TBI is similarly applicable in the adult population due to challenges with controlling heterogeneity and an increased number of required isocenters with increasing patient size; however, there is currently a paucity of literature for the adult patient population.

An important consideration for VMAT‐TBI is the longitudinal travel extent of the treatment couch, which determines the maximum patient height that can be treated in the head‐first‐supine (HFS) orientation. For patients taller than approximately 115 cm, treatment requires both HFS and feet‐first‐supine (FFS) orientations to ensure full‐body coverage. To streamline the transition between these orientations, some institutions utilize a rotating couch top attached to the standard linear accelerator (linac) and CT simulator couches during simulation and treatment.^5^, ^6^, ^7^, ^8^ While commercially available systems (e.g., System TBI STEP from IT‐V (Innsbruck, Austria)^7^ equilibrium rotating patient platform from CDR systems (Calgary, Alberta, Canada)) have been in use outside the United States for several years (although at the time of the publication of these works, commercial products did not exist), many institutions within the US have developed in‐house solutions to address this need.^5^, ^6^, ^7^, ^8^ In this work, we describe the design and technical components of “Spinning Manny,” an in‐house developed rotational platform for streamlining VMAT‐TBI treatments at Stanford University. The design is shared publicly via GitHub using the GNU general public license (https://github.com/lawrie83/VMAT‐TBI‐SpinningManny).

## 2 MATERIAL AND METHODS

VMAT‐TBI was implemented clinically at Stanford University in October 2019. Design, production, and testing of the Spinning Manny platform were included in the preparatory and commissioning stages of VMAT‐TBI.

### 2.1 Spinning Manny design

**FIGURE 1:**
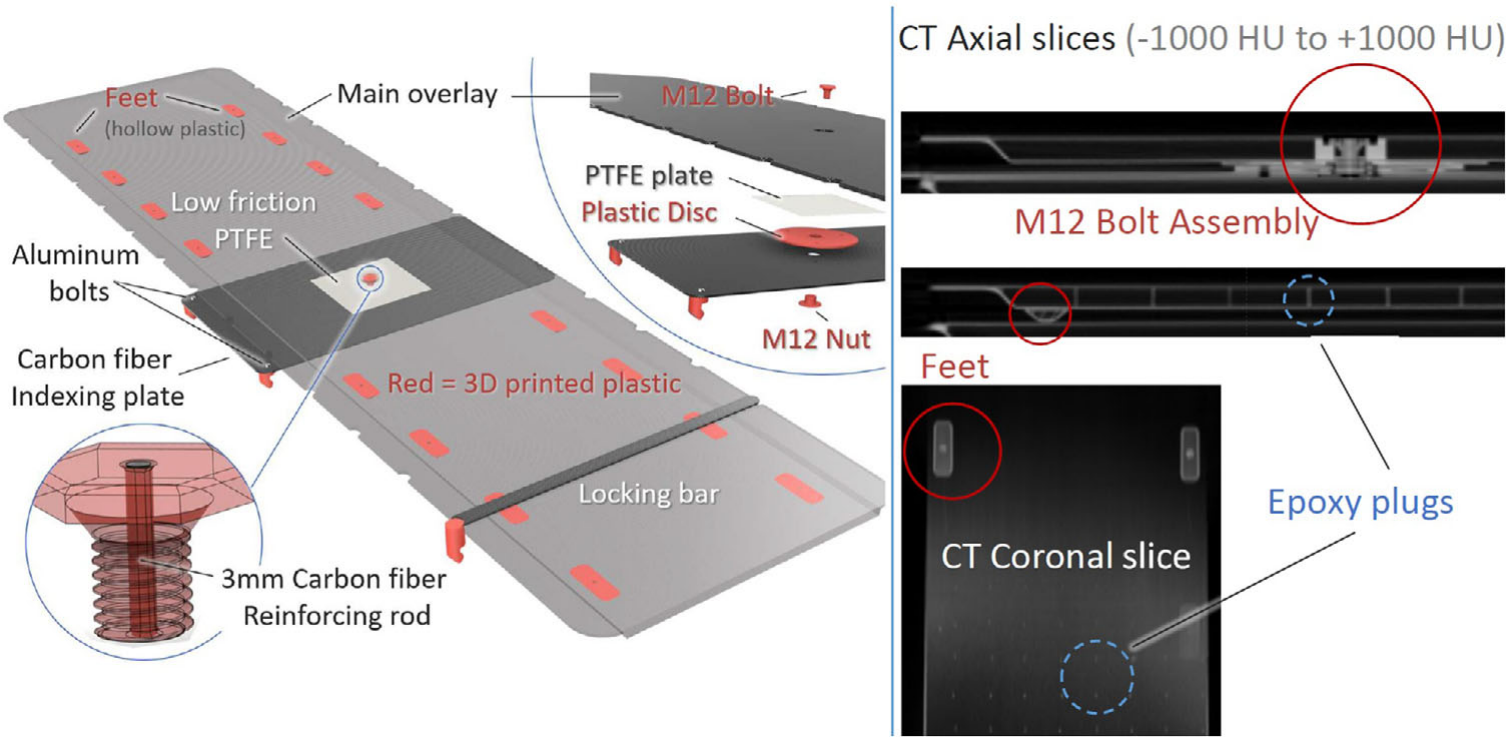
CAD and CT images of the Spinning Manny overlay system, which comprises of a main carbon fiber overlay, a smaller carbon fiber indexing plate, a locking bar, a central pivot, and several smaller pieces. The CT slices shown on the left side contain the main components.

The Spinning Manny platform consists of a 216 × 52.5 × 1.5 cm^3^ foam‐core carbon fiber shell, which rotates about a pivot location that is 100 cm from the inferior end and 126 cm from the superior end (Figure 1). The carbon fiber wall thickness is 1.5 mm (density ∼1.6 g/cm^3^) and the foam core is 12 mm thick (density ∼0.13 g/cm^3^). This platform is bolted to a carbon fiber base plate (57 × 32 × 0.3175 cm^3^) which indexes to four points on the main treatment table at positions H0 and F2. The indexing locations on the Spinning Manny match those of the original treatment table (14 cm index spacing). Two thumb bolts are used to secure the base plate to the couch top and two additional index pegs are used to securely lock the platform at each end of the main treatment table to prevent any motion after alignment. The centerline and pivot plane are clearly marked to guide therapists in positioning the patient relative to the table.

The overlay board and carbon fiber base plate were machined by Hillsdale Composites (El Cajon, CA). The default manufacturing of the overlay board results in 3 mm diameter epoxy cylinders every 5 cm inside the foam core for support. For a higher cost, these epoxy cylinders can be eliminated. This additional expense was deemed unnecessary for the current VMAT‐TBI application. To enable dissemination, maintenance, and adaptation, the smaller parts of the Spinning Manny were designed to be 3D‐printed. The bolt securing the Spinning Manny to the indexing plate is critical as high fracture toughness is needed for durability while simultaneously minimizing radiation attenuation. A large diameter (12 mm) nylon bolt reinforced along its central axis with a 3 mm diameter carbon fiber rod was used for the central bolt. The addition of the carbon fiber was found to increase the shear strength by a factor ∼40 as the continuous carbon fibers have higher fracture toughness than nylon alone. The design files for all these components are publicly shared.^9^

### 2.2 Secure indexing system

**FIGURE 2:**
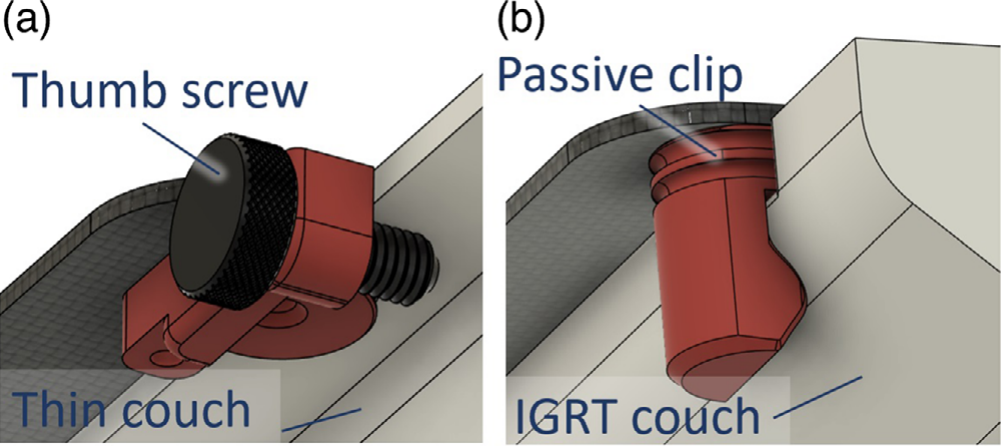
Close‐up of the two types of secure indexing for different couch shapes. (a) For thin couches such as the Calypso kVue couch top, and (b) for the Varian IGRT carbon fiber couch top.

The indexing points were designed with two variations: a screw lock mechanism for thinner couch tops and a clip‐on design for compatibility with thicker, rounded Varian IGRT carbon fiber couch tops (Varian Medical Systems, Palo Alto, CA). The technical drawings for the index locking system are shown in Figure 2.

### 2.3 Spinning Manny commissioning tests

#### 2.3.1 Weight, fit, and positioning reproducibility tests

A healthy adult volunteer (weight ∼82 kg) plus 55 kg of Solid Water (Gammex Technology, Sun Nuclear Corporation, Melbourne, FL) was placed on the Spinning Manny to evaluate its durability. The 55 kg of Solid Water was split into two equal stacks, each measuring 30 × 30 × 30 cm, and were placed at the extreme ends of the Spinning Manny platform. The platform was then rotated with the assistance of two people at the head and two at the foot of the couch top. Three to four people aid in the rotation of the Spinning Manny as a precautionary measure, especially since many patients have intravenous lines or an audio‐visual assistant therapeutic ambience in radiotherapy system that requires careful attention during rotation. The pivot and indexing system were inspected for stress and fracture. The 27 kg Solid Water stacks added significantly more stress and torque to the pivot joint than a patient of the same weight as the stacks were positioned approximately 0.8–1.1 m from the pivot joint, whereas the patient weight would be spread uniformly over the platform. The indexing devices and footlocker were inspected for fit before use. The Spinning Manny was also tested for reproducibility of isocenter alignment following rotation. The approximate pelvis isocenter position was marked on the platform at 19.0 cm superior to the pivot point. The platform was rotated 180° and the position of the marked pelvis isocenter was verified.

#### 2.3.2 Dosimetric tests

As described in the previous sections, the Spinning Manny was designed to have sufficient strength while minimizing MV x‐ray attenuation. The system was scanned on a Siemens Biograph PET/CT scanner (Siemens, Munich, Germany), different regions on the resulting image were contoured, and the average HU of these regions was measured. The model for the Spinning Manny was created in the Eclipse treatment planning system (Varian Medical Systems, Palo Alto, CA) with averaged HU assignments to be used for treatment planning.

Attenuation measurements of the Spinning Manny were made using the recommendations of the American Association of Physicists in Medicine (AAPM) Task Group (TG) report 176.^10^ The measurement setup consisted of a 30 × 30 × 20 cm Solid Water stack with a farmer‐type ionization chamber placed at 10 cm depth using an SAD of 100 cm for beam energies of 6 and 10 MV. Charge measurements were made at multiple opposing gantry angles ranging from 0° to 300° in 10° increments to calculate the Spinning Manny beam attenuation for various angles of incidence. Measurements were performed with the Spinning Manny indexed on the treatment couch such that the Solid Water phantom was positioned off the end of the treatment couch (i.e., attenuation of Spinning Manny only). One additional attenuation measurement set was performed for a beam energy of 10 MV with the Solid Water phantom positioned over the treatment couch to determine the combined attenuation of Spinning Manny and the Qfix kVue IGRT couch top (CQ Medical, Avondale, Pennsylvania, USA).

End‐to‐end testing was performed using an anthropomorphic Rando phantom (Radiology Support Devices, Gardena, CA) with an adjacent Solid Water phantom. Dosimetric verification of the platform model was evaluated by delivering test plans on the phantom, where measured and calculated axial and coronal dose distributions were compared. The axial dose distribution was measured in the lung region, whereas the coronal dose distribution was measured in the VMAT/AP/PA junction region. Dose distributions were measured using calibrated Gafchromic EBT3 film (Ashland Corporation, Bridgewater, NJ). Further dosimetric validation of the platform model was performed using the IROC TBI phantom using TLDs, which is required for credentialing on the COG ASCT2031 trial^11^ for VMAT‐TBI.

### 2.4 Clinical implementation of Spinning Manny

#### 2.4.1 CT Simulation

CT simulation was performed on a Siemens Biograph CT scanner by acquiring a full‐body scan of the patient with an extended field of view and a slice thickness of either 5 mm (adult patients) or 3–4 mm (pediatric patients). Extended field of view is always employed during VMAT‐TBI scanning to capture all immobilization devices, including the rotational platform and vak‐bag. The CT reference ball bearings (BBs) are typically positioned on the lateral sides of the vak‐bag, while additional BBs marking the 100 cm couch longitudinal position are placed at the most lateral points of the rotating platform. Thus, the extended field of view ensures that all of these components are included in the scan. Additionally, the Varian TPS Eclipse system typically encounters an issue when the entire body is not captured in the scan, which may create a gap in the body structure and hinder dose calculations for beams passing through that gap. For patients height under 115 cm, the Spinning Manny is not required as the patient can be treated entirely in the HFS orientation. For patients taller than 115 cm, simulation and treatment should be performed on the Spinning Manny to streamline the transition from the HFS to FFS orientations. Of note, the Siemens Biograph PET/CT scanner is capable of performing full‐body scans in a single session, eliminating the need for separate HFS and FFS scans and concatenation; however, some other CT scanners may require separate HFS and FFS scans and concatenation.

During simulation, the patient is immobilized in HFS position in a whole‐body vacuum bag on an indexing bar with arms placed tightly at the sides of the body with palms touching the sides of the legs. The patient's head rests on an Accuform cushion, immobilized in a thermoplastic mask, and the whole‐body vacuum bag is carefully molded around the shoulders. The positioning of the patient on the Spinning Manny is such that the HFS portion of treatment encompasses at least 110 cm of the upper body (i.e., maximize the extent of the patient that can be treated in the HFS orientation). The selection of patient positioning on the Spinning Manny impacts the location of the matchline or pivot plane. The ideal match line position is chosen for two key reasons: (1) geometric—it allows for the fit of 3 isocenters of 40 × 40 fields with sufficient 2–5 cm overlap within ∼110 cm distance, ensuring optimal coverage; (2) anatomic—the match line is ideally placed at the knees or mid‐shin for younger patients, or at the mid‐thigh for taller patients. This match area avoids critical organs at risk, ensuring they are not overdosed during treatment.

#### 2.4.2 VMAT‐TBI treatment planning

**FIGURE 3:**
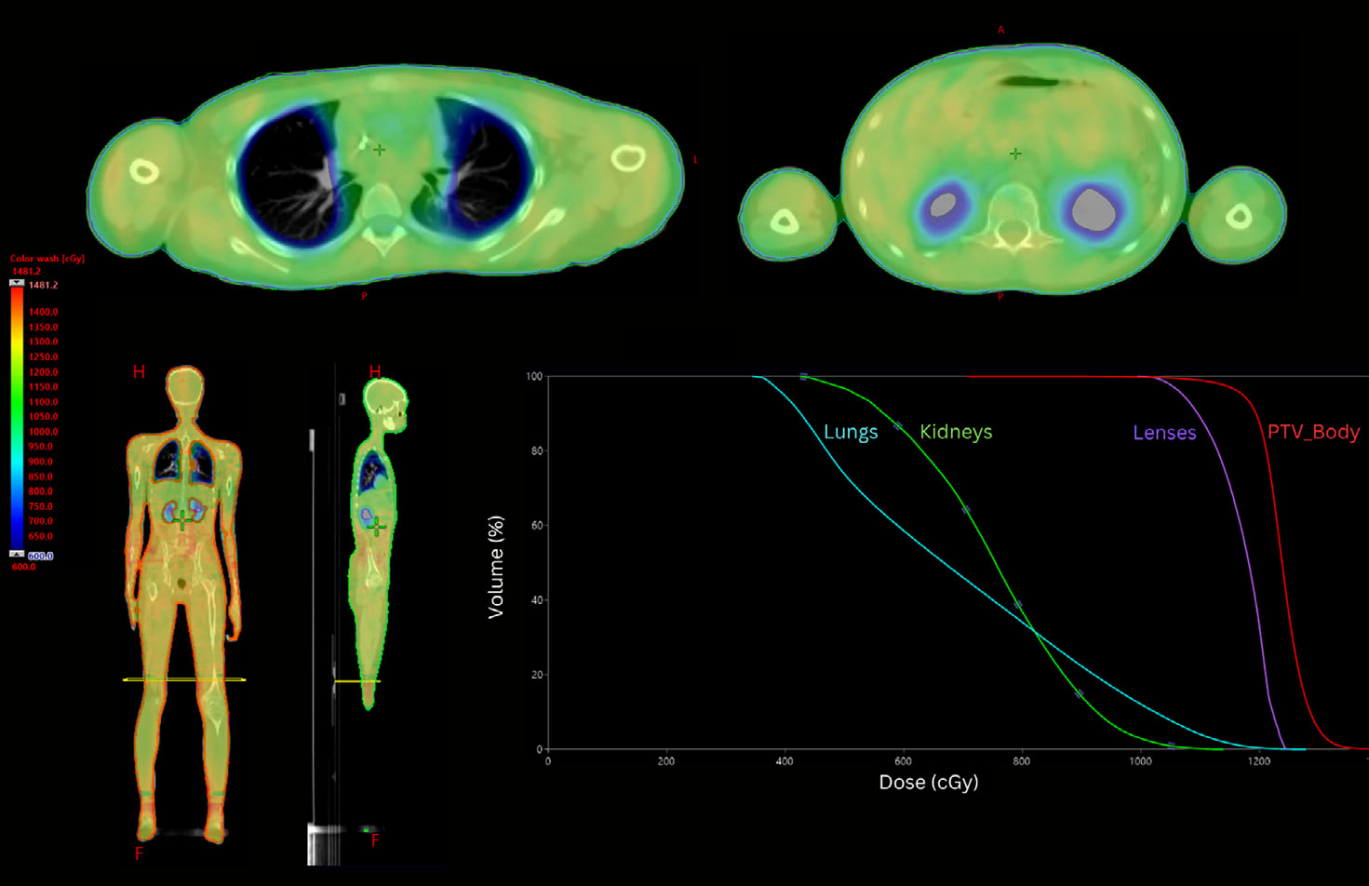
Dose volume histogram (right bottom) and dose distribution in the axial (top), coronal, and sagittal (left bottom) planes for a pediatric patient treated with 12 Gy in six fractions using the Stanford VMAT‐TBI technique. The dose cloud is thresholded at 50% of the prescription dose. The matchline contour between HFS VMAT portion of the plan and FFS AP/PA portion of the plan is denoted in yellow. FFS, feet‐first‐supine; HFS, head‐first‐supine; VMAT‐TBI, Volumetric Modulated Arc Therapy‐total body irradiation.

The treatment couch overlay and the Spinning Manny platform are inserted as couch support structures and are taken into account during optimization and dose calculation. For patients simulated on the Spinning Manny, a rectangular “matchline” contour (a rectangular contour on a single axial slice) is outlined in the center of the Spinning Manny bolt found on the sagittal view. This matchline contour is then used by open‐source auto‐planning scripts^8^, ^12^ to determine isocenter placement and separate the target into an upper VMAT portion and a lower AP/PA portion. The pelvis and upper leg isocenters are set equidistant from the matchline to simplify patient setup following rotation from the HFS to FFS orientations. An example VMAT‐TBI treatment plan dose distribution with associated DVH is shown in Figure 3.

#### 2.4.3 Pre‐treatment imaging, in vivo dosimetry verification, and delivery of VMAT‐TBI

Prior to treatment, patients are carefully aligned to the vacuum bag, which is then aligned to the Spinning Manny based on the marks from simulation. Although patients are treated on couches with six degrees of freedom, the couch rotations are disabled during imaging and treatment of VMAT‐TBI to ensure accurate matching of fields throughout the length of the whole body. Delivery of VMAT‐TBI involves aligning the patient to the chest isocenter, performing kV‐pair and CBCT, applying online alignment corrections, and then treating. The longitudinal shift is then applied to move to next isocenter, an MV open port (attached to the first treatment arc) is then acquired, isocenter positioning is verified allowing <5 mm residual shift in the lateral direction only, and then the isocenter is treated. If shifts are greater than 5 mm, therapists must then perform manual adjustments to ensure accurate alignment. This process is repeated for every subsequent isocenter. After the pelvis isocenter is treated (with the pelvis plan still moded‐up), the gantry is rotated to 0°, the couch is lowered, and the Spinning Manny is rotated 180° with 1–2 people holding the platform on both sides (see video in Supporting Information). Following rotation, the ‘auto‐go’ button is pressed on the couch or pendant and the couch is positioned to the same longitudinal and vertical parameters as the pelvis isocenter. The therapists then shift the couch laterally to the opposite sign to account for the patient orientation change to FFS.

In vivo dosimetry was performed using optically stimulated luminescence detectors (OSLDs) placed on the patient in the VMAT/AP/PA junction region to ensure proper dosimetric matching for the first 35 patients treated at our institution. Efficiency improvements using the Spinning Manny were assessed by evaluating treatment records in the RT Summary workspace for 20 treated fractions of VMAT‐TBI. This data was collected both at Stanford University, where the Spinning Manny system was used, and at the University of Alabama at Birmingham (UAB), which does not utilize a rotational platform. At the University of Alabama at Birmingham, where the Spinning Manny rotational platform is not used, the patient must first be fully unloaded from the treatment/simulation couch, then the immobilization device is rotated and re‐indexed, and the patient is subsequently re‐setup and aligned in the immobilization device. All other steps are the same between the two institutions. In addition to improvements in efficiency, requiring the patient to be fully unloaded from the couch has several other caveats, including safety issues (these patients are often frail and requiring them to get up/lie down multiple times is not ideal) and positioning uncertainty (setting up the patient multiple times increases the positioning uncertainty of the patient).

#### 2.4.4 Periodic testing

The commissioning tests of the Spinning Manny were conducted when the system was initially installed to establish baseline performance. To ensure continued optimal function, annual QA procedures have been implemented since then. These tests include comprehensive checks on mechanical alignment, the integrity of the overlay components, and system performance, all aimed at confirming that the couch overlay remains as effective as it was during the initial commissioning phase. Additionally, end‐to‐end tests are performed to verify the system's overall functionality and ensure that it continues to meet the required standards for treatment accuracy and safety.

## 3 RESULTS

Prior to 2024, only one Spinning Manny platform was manufactured. In 2024, four identical Spinning Manny platforms were manufactured using the updated design that preserved the physical dimensions of the platform but improved the sturdiness: one for CT simulation and three for the Varian TrueBeams that are used for VMAT‐TBI treatment at Stanford Radiation Oncology.

### 3.1 Spinning Manny commissioning tests

#### 3.1.1 Weight, fit, and positioning reproducibility

The Spinning Manny platforms can support a weight of 159 kg (136 kg test weight + 23 kg accounting for placement of the Solid Water at the extremes of the platform). For comparison, for the unmodified Varian table, older versions of the Varian six degrees of freedom couch support up to 159 kg, and the limit has increased on the newer version to 200 kg. Further testing and verification is needed for weights greater than 159 kg. Magnets and a tether were added to the footlocker to ease clinical workflow and improve safety. Isocenter position reproducibility was observed to be within 1 mm after rotation of the platform.

#### 3.1.2 Dosimetric tests

The dosimetric properties of the Spinning Manny components are shown in Table 1. Based on the acquired CT scan, a four‐component support structure model was created using the averaged CT numbers: Spinning Manny Plate (‐250 HU, 0.76 g/cm^3^), Spinning Manny CFRP (‐550 HU,0.448 g/cm^3^), Spinning Manny Teflon (990 HU, 2.2 g/cm^3^), and Spinning Manny Bolt (‐150 HU, 0.868 g/cm^3^).

**TABLE 1:** Attenuation measurements following the TG176 protocol (employing open beams at 180° opposing angles), through the Spinning Manny overlay for beam energies of 6 and 10 MV.

Gantry angle (°) degrees	Spin‐Man 6 MV	Spin‐Man 10 MV	Qfix + Spin‐Man 10 MV
300	4.3%	3.4%	5.2%
310	3.3%	1.9%	4.9%
320	2.4%	2.0%	4.1%
330	1.8%	1.6%	3.4%
340	1.9%	1.2%	3.1%
350	1.8%	1.2%	3.0%
0	1.3%	1.0%	2.8%
Average	2.1%	1.6%	3.4%

*Note*: Additional measurements through both the Qfix kVue couchtop and Spinning Manny also shown (right column).

The Spinning Manny attenuation measurements are shown in Table 1. Maximum and minimum attenuation occurred for gantry angles of 300° and 0°, respectively, for all beam energies. End‐to‐end testing with the Rando phantom (Radiology Support Devices, Gardena, CA) and axial film measurements in the lung area showed a 3%/2 mm Gamma passing rate of 96.9%. End‐to‐end testing with coronal film measurements in the VMAT/AP/PA junction region following platform rotation showed 5%/3 mm Gamma passing rate of 92.6% and 3%/5 mm Gamma passing rate of 90.1%. A looser gamma analysis criteria was used for evaluation in the junction region due to the additional uncertainty introduced by rotating the phantom on the platform. The measurement results for the IROC VMAT‐TBI credentialing phantom for the COG ASCT2031 clinical trial are shown in Table 2. All TLD‐measurements outside of lungs in regions of homogeneous dose showed agreement within 3% between the planned and measured dose. Inside the lungs, agreement within 7% was observed due to the higher dose gradients caused by the lung sparing. Per IROC, the recommended tolerances for the measurements are ±15% for all locations except the midline location of umbilicus, which has a tolerance of ±5%.

**TABLE 2:** IROC VMAT‐TBI credentialing results for the COG ASCT2031 trial.

Location	Institution reported dose (cGy)	TLD measured dose (cGy)	Measured/reported
Mid‐brain	309	314	1.02
Neck	311	321	1.03
Mid‐mediastinum	312	311	1.00
Umbilicus	307	310	1.01
Mid‐pelvis	310	307	0.99
Superior‐lung‐right	166	165	1.00
Mid‐lung‐right	67	69	1.03
Inferior‐lung‐right	155	144	0.93
Superior‐lung‐left	150	156	1.04
Mid‐lung‐left	94	99	1.06
Inferior‐lung‐left	117	113	0.96

*Note*: Tolerances for agreement are ±15% for all locations except the umbilicus, which has a tolerance of ±5%.

Abbreviations: TLD, thermoluminescent dosimeters; VMAT‐TBI, Volumetric Modulated Arc Therapy‐total body irradiation.

### 3.2 Clinical implementation of Spinning Manny

In total, 136 patients underwent VMAT‐TBI treatment at our institution from October 2019 to February 2025. Median patient age was 14 years (range, 1–64 years), average patient height was 150.4 ± 28.6 cm, and average width was 41.8 ± 8.6 cm. The median number of isocenters was 5 (range, 3–7). Spinning Manny was successfully used for all patients requiring HFS and FFS treatment (113 patients out of 136, 83%). For the first 35 patients, the average in vivo dose measurement on the matchline between the VMAT and AP/PA portions of the treatment indicated patient setup was reproducible (96.1 ± 5.5% relative to prescription dose). In addition, the total treatment time for the same 35 patients was 47.5 ± 9.5 min, including patient setup and beam‐on time.

The comparison of isocenter transition times revealed notable differences between the institutions using the Spinning Manny system and those relying on manual repositioning. To perform a fair comparison between the two institutions (as the patient populations and treatment vault designs are different), the time to transition between isocenters was calculated for 20 patients. All of the extracted transition times were normalized to the average time to transition between isocenters that do not require a rotation from HFS to FFS orientation (e.g., mean time of head—chest, chest—abdomen, abdomen—pelvis, and upper leg—mid/lower leg transitions). At Stanford University, the average time for transitioning from the HFS to FFS position was 1.6 times longer than other isocenter transitions. In contrast, at the University of Alabama at Birmingham, which does not use a rotational platform and relies on manual repositioning of the patient, the transition time from HFS to FFS was 2.6 times longer compared to other isocenter transitions. At the University of Alabama at Birmingham, the patient is required to be unloaded from the treatment couch, the immobilization device re‐indexed, and the patient is re‐set up in the immobilization device following transition to the FFS orientation, which, in addition to adding treatment time, also adds unnecessary uncertainty in patient setup/positioning.

### 3.3 Periodic testing

Annual quality assurance testing confirmed that the Spinning Manny system maintained its mechanical and dosimetric performance over time. Mechanical alignment remained within 1 mm of baseline measurements, and no degradation of overlay structural integrity was observed after repeated clinical use. End‐to‐end evaluations consistently demonstrated gamma passing rates >95% (3%/2 mm), verifying sustained treatment accuracy.

## 4 DISCUSSION

Various groups have described the use of rotational tabletops for VMAT‐TBI treatments in the literature (Table 3). Springer et al.^5^ described a vacuum mattress placed into a custom wooden box to ensure a stable mattress from the start of simulation until the end of treatment. Eight patients were treated first in the HFS position, then rotated 180° to be subsequently treated in the FFS position, with accurate delivered treatment doses verified with MOSFETs. Ouyang et al.^6^ designed a custom indexed rotatable immobilization system (IRIS), which is comprised of a rotational platform, a patient‐immobilizing body frame, and beam‐spoiler attachment. Similar to the Spinning Manny, IRIS locks onto the CT and linac couches with a top plate that is supported and secured with a rotating disc that enables the plate to rotate around a pivot point. Losert et al.^7^ described the use of a rotatable carbon fiber tabletop facilitating a HFS position to a FFS position that can be mounted on the CT or linac couch top. The rotational tabletop design described by Losert et al.^7^ was later commercialized and has been available in Europe for many years and only recently brought to market in North America (System TBI STEP from IT‐V (Innsbruck, Austria)). In addition, other commercial solutions are also now available in North America (e.g., equilibrium rotating patient platform from CDR Systems (Calgary, Alberta, Canada)). Similar to this work, these studies utilize custom rotational platforms for multi‐isocenter VMAT‐TBI treatments to overcome couch length limits and report robustness in treatment precision and reproducible dose delivery.

**TABLE 3:** VMAT‐TBI techniques described in the literature.

Reference	TPS/TDS	Rotational table	Bolus	Technique/no. of iso's	Dose rate MU/min	PTV *D*_max_	Lung *D*_mean_	Kidney *D*_mean_	Other organs spared	Planning/treatment time	Imaging	QA/In vivo
Springer 2016^5^	Eclipse AAA v10/Varian	In‐house made wooden box	Yes, whole body	VMAT/9–15	600	130% at 1.6% of volume	9.6 (80%)	–	Prior RT, kidney for select patients	36 h/2 h	kV each iso	ArcCheck MOSFET
Ouyang 2017^6^	Pinnacle/Varian TrueBeam	IRIS (in‐house developed)	Yes, for legs only	VMAT with AP/PA/5–7	40/50–600	133% *D*_max_	Lung 1 cm 9 Gy (75%)	–	–	NA/1 h 20 min	CBCT chest iso, no imaging after	Film, IC in solid water/OSLD
Tas 2018^14^	Monaco v5.11/Electa Versa HD	No	No	VMAT/6–7	50–600	HI = 1.16	9.6 Gy (80%)	9.6 Gy (80%)	Lens	NA/55 min for adult, 35 min for pediatric	CBCT each iso, surface guidance	DVH based using transmission detector
Losert 2019^7^	Monaco v5.11/Elekta	In‐house developed, commer. available in Europe	No, 2.5 mm flash	VMAT/6–7	–	–	<10 Gy (83%)	–	–	NA/57 min	CBCT for head, abdomen, and knees for fx 1, surface imaging	–
Blomain 2020^15^, Simiele 2021^12^, Kovalchuk 2022^8^	Eclipse AAA v15.6/Varian/TrueBeam	In‐house developed	No, 5 mm flash	VMAT with AP/PA/3–5	100–600	*D*1cc <120%, V110% <5%	Lung 57.6%, lung 1 cm 40.8%	70%	Lens *D*max<90%, for benign: gonads, brain, thyroid, lens; For select: prior RT, liver, heart	4–5 h/47.5 min	CBCT chest iso, MV port for each subsequent iso	EPID portal dosimetry, Mobius3D/OSLD
Guo 2021^13^	Pinnacle v16.2/Varian TrueBeam	No	No	VMAT with AP/PA/6–8	200–600	ALARA for V110	Goal of <10 Gy (83%)(adult), <8 Gy (67%) pediatric	Goal of <50%	–	12 h/1 h	CBCT at chest iso, kV/kV for each subs. iso	EPID portal dosimetry/OSLD
Teruel 2021^16^	Eclipse AAA v15.6/Varian TrueBeam	No	No, 2 cm flash sup of head	VMAT with AP/PA/7	600	*D*_2cc_ <130%	<8 Gy (12 GyRx), <9 Gy (13 GyRx)	<11 Gy	–	N/A/1 h 15 min	N/A	ArcCheck, EPID portal dosimetry/OSLD
Stanley 2021^17^	Eclipse AAA v16.1/Varian TrueBeam	No	Yes, 1 cm over tibia	VMAT with AP/PA 4–6	40–300	*D*max <16 Gy	Lung 1 cm <8 Gy (75%)	–	–	NA/1 h 15 min	kV/kV each iso	EPID portal dosimetry, Mobius 3D, IC in solid water, OSLD
Keit 2023^18^	Raystation 11A/Varian TrueBeam	No	No	VMAT with AP/PA 4–7	N/A	ALARA	<7.5 Gy	<7 Gy	Heart <6.5 Gy, breast <5 Gy	N/A	N/A	SunCHECK (second MU calc. and IMRT QA)

Abbreviations: TLD, thermoluminescent dosimeters; VMAT‐TBI, Volumetric Modulated Arc Therapy‐total body irradiation.

Compared to other previous works that have described rotational tabletops for use with VMAT‐TBI, the current study is unique for several reasons. First, the design and build instructions for Spinning Manny are shared open‐source via GitHub under the GNU General Public License.^9^ Second, dissemination of the Spinning Manny design is part of a larger initiative from our group to encourage transition from conventional to modern TBI treatment techniques in the radiation oncology community worldwide. In addition to Spinning Manny, our group has also shared automated treatment planning scripts for the Eclipse treatment planning system for VMAT‐TBI (https://github.com/esimiele/VMAT‐TBI‐CSI), which were designed to work with the Spinning Manny tabletop using the Stanford VMAT TBI technique.^8^ Third, the cost to build a Spinning Manny is significantly less than comparable commercial options. One common obstacle in transitioning from conventional to modern TBI techniques is securing equipment specific to VMAT‐TBI. In the authors’ experience, it can be difficult to convince hospital administration to purchase equipment that will only be used for a handful of patients each year, which is dependent on hospital transplant volume and patient population characteristics (i.e., height). In addition, reimbursement from VMAT‐TBI is generally lower compared to other forms of VMAT treatment, further discouraging significant resource investment from administration. Therefore, reducing costs as much as possible increases the likelihood of securing equipment for VMAT‐TBI with minimal resistance. The estimated cost of commercial tabletop solutions is generally greater than US$ 35 000 (based on quotes obtained in 2024), whereas the cost to build a Spinning Manny is less than US$ 5000 (2024), where a majority of the cost is from manufacture of the overlay board and carbon fiber plate (∼75% of total cost). The low cost of Spinning Manny provides a low barrier of entry to securing a rotational tabletop for VMAT‐TBI treatments, thus increasing access to this modern TBI treatment technique.

The development and implementation of the Spinning Manny Indexed Overlay System for VMAT‐TBI at Stanford University has significantly streamlined the treatment process for patients requiring both HFS and FFS positioning. The introduction of this system addressed a critical limitation in the VMAT‐TBI workflow by ensuring efficient and reproducible patient repositioning while maintaining dosimetric accuracy. The results of this work demonstrate the Spinning Manny enhances treatment precision and consistency, reducing the complexity associated with manual patient repositioning. Despite these advancements, further refinements are warranted and limitations are present. For example, under the European Union's Medical Device Regulation, the use of self‐built medical devices may not be produced if an equivalent device is available on the market, limiting the use of the Spinning Many open‐source design and build instructions for institutions in the European Union. In terms of design, the minor discrepancy in indexing dimensions, which was rectified with shims, highlights the importance of precise manufacturing tolerances. Additionally, while the Spinning Manny has demonstrated adequate weight support, further stress testing for higher‐weight patients would be beneficial to ensure robustness across all clinical scenarios. Moreover, integration of additional safety features such as automated locking mechanisms could enhance ease of use and safety.

## 5 CONCLUSION

The Spinning Manny Indexed Overlay System represents a significant innovation in VMAT‐TBI treatment, addressing logistical and dosimetric challenges associated with patient repositioning. Its mechanical stability, low attenuation, and high isocenter reproducibility support its clinical reliability, with successful implementation across patients of varying sizes and ages. The successful implementation of Spinning Manny at Stanford University provides a model for other institutions seeking to adopt VMAT‐TBI, particularly as the transition away from traditional 2D techniques continues. Future work should focus on expanding weight tolerance, optimizing manufacturing precision, and exploring additional automation features to further improve clinical workflow and patient safety. By sharing our design and implementation experience, we aim to facilitate the broader adoption of VMAT‐TBI globally, improving access to advanced TBI techniques for patients undergoing HSCT.

## AUTHOR CONTRIBUTIONS

Lawrie Skinner: Conception and design; acquisition of data; drafting and reviewing manuscript. Eric Simiele: Acquisition of data; analysis and interpretation; drafting and reviewing manuscript. Zi Yang: Acquisition of data; analysis and interpretation; drafting and reviewing manuscript. Caressa Hui: Acquisition of data; analysis and interpretation; drafting and reviewing manuscript. Ignacio Romero: Acquisition of data; analysis and interpretation; drafting and reviewing manuscript, Michael Binkley: Analysis and interpretation; drafting and reviewing manuscript. Richard Hoppe: Analysis and interpretation; drafting and reviewing manuscript. Susan M. Hiniker: Conception and design; drafting and reviewing manuscript. Nataliya Kovalchuk: Conception and design; acquisition of data; drafting and reviewing manuscript.

## CONFLICT OF INTEREST STATEMENT

The authors declare no conflict of interest.

## Supporting information



Supporting Information

## Data Availability

The data that support the findings of this study are available on request from the corresponding author.
